# Assessment of Appearance-related Questions About Breast Reconstruction Generated by Chat Generative Pre-trained Transformer

**DOI:** 10.1097/GOX.0000000000006625

**Published:** 2025-03-21

**Authors:** Xiomara T. Gonzalez, Margaret S. Roubaud, Mark V. Schaverien, Rene D. Largo, Christopher S. Parham, Ashleigh M. Francis, Tzuan A. Chen, Aubri S. Hoffman, Ryan M. Dickey, Mia K. Markey, Gregory P. Reece

**Affiliations:** From the * Department of Electrical and Computer Engineering, The University of Texas at Austin, Austin, TX; † Department of Plastic Surgery, The University of Texas MD Anderson Cancer Center, Houston, TX; ‡ Department of Psychological, Health, and Learning Sciences, HEALTH Research Institute, University of Houston, Houston, TX; § Department of Biomedical Engineering, The University of Texas at Austin, Austin, TX; ¶ Department of Imaging Physics, The University of Texas MD Anderson Cancer Center, Houston, TX.

## Abstract

**Background:**

Previous studies have explored the ability of artificial intelligence (AI) tools based on large language models, such as Chat Generative Pre-Trained Transformer (ChatGPT), to answer patient questions about breast reconstruction. In this study, we assessed the quality of questions generated by ChatGPT for breast reconstruction patients to ask their providers.

**Methods:**

ChatGPT was prompted to generate appearance-related questions representative of what patients might ask during breast reconstruction consultations. As a benchmark, a comparison group of questions from credible online sources was compiled. Blinded to the source, surgeons assessed the quality of questions in terms of their acceptability, contribution to the informed consent process, and contribution to the shared decision-making process. Surgeons were also asked to report their agreement on whether questions were generated by an AI tool.

**Results:**

Experienced reconstructive surgeons rated ChatGPT-generated questions about appearance-related outcomes of breast reconstruction as acceptable (15 of 16), likely to positively contribute to the informed consent process (15 of 16), and likely to positively contribute to the shared decision-making process (16 of 16). These ratings were comparable to those for benchmark questions. Surgeons did not readily recognize questions as being AI-generated. Differences in surgeon assessments were most pronounced regarding the perceived potential for the questions to contribute to the informed consent process.

**Conclusions:**

The quality of ChatGPT-generated questions related to appearance concerns is comparable to that of questions sourced from reputable online websites. Patients may benefit from discussion with their providers about best practices for using AI tools in preparation for consultations.

**Takeaways**

**Question:** What is the quality of appearance-related questions generated by Chat Generative Pre-Trained Transformer (ChatGPT) for patients who are planning to undergo breast reconstruction to ask their providers?

**Findings:** The quality of ChatGPT-generated questions regarding appearance-related concerns is comparable to questions sourced from reputable online websites. Surgeons did not readily recognize ChatGPT questions as being artificial intelligence (AI)–generated.

**Meaning:** With the advancement of publicly accessible AI tools, patients may benefit from discussions with their providers about the best practices for leveraging AI tools for consultation preparation.

## INTRODUCTION

Approximately 158,000 women undergo breast reconstruction annually in the United States as part of their breast cancer treatment.^1^ Forty percent of women who have a breast reconstruction procedure rate its outcome as worse than expected, primarily citing appearance-related concerns.^2^ Often, these misaligned expectations stem from gaps in patients’ understanding of how breast reconstruction will alter their appearance.^2–4^ Unmet expectations about breast reconstruction are associated with increased body image concerns, decision regrets, mental health disorders, and reduced overall quality of life.^5,6^

Patients who ask questions are more likely to have their information needs met.^7^ Patients with breast cancer who are better prepared for discussions with their providers report fewer communication barriers and confidence with decision-making.^8^ Likewise, health professionals (eg, breast surgeons and nurses) are more satisfied with patient-provider communications when patients ask questions during consultations.^7,8^ Appropriately prepared patient-physician dialogue reduces decision unease for patients seeking breast reconstruction.^7^

Previous research has explored improving communication between patients with breast cancer and their providers by having patients draft questions before consultation through 2 avenues: question listing and question prompt sheets.^9,10^

Question listing involves patients creating their own list of questions,^11^ sometimes with assistance of consultation planning personnel.^12,13^ An advantage of patient-generated questions is that they emphasize the patient’s interests. However, for many patients with breast cancer, the urgency to receive immediate treatment leads to questions focused on the risk of surgical complications rather than on their goals and expectations for breast reconstruction.^14^ Some patients also report that generating their own question list is mentally taxing because they do not understand their condition well enough to advocate for their desired reconstruction options,^15^ leading to guilt for not asking the “right” questions.^16^

Question prompt sheets are curated lists of questions from sources, such as breast cancer organizations or medical centers that patients can select from to discuss with their healthcare team.^9,10,17–19^ An advantage of question prompt sheets is that they can encourage patients to consider questions they may not have thought to ask their provider.^10,20,21^ However, patients may perceive question prompt sheets as less valuable if they primarily focus on *cognitive* information needs—concerning what is happening to the patient and the disease—rather than addressing *affective* information needs, encompassing patients’ concerns and fostering a sense of understanding from providers.^22,23^

Over the last few years, artificial intelligence (AI) tools based on large language models (LLMs) have become widely available to the public, primarily through conversational interaction tools, such as Chat Generative Pre-Trained Transformer (ChatGPT). Recently, studies have investigated the quality of *answers* generated by LLMs to questions about breast cancer care,^24–26^ including breast reconstruction topics.^27,28^ For example, Liu et al^27^ found that ChatGPT provided high-quality answers to 10 common questions about breast reconstruction, although 40% of surgeons surveyed expressed reluctance to endorse ChatGPT for patient education. Prior work has also demonstrated efficiency gains from using LLMs to answer questions. For instance, Hung et al^28^ reported a significant reduction in the time required to generate breast reconstruction patient education materials using ChatGPT; however, the accuracy of the content was low, and the readability scores were higher than recommended for patients. As yet, no study has investigated the quality of *questions* about breast reconstruction generated by LLMs.

The purpose of this study was to assess the quality of appearance-related questions about breast reconstruction generated by ChatGPT, intended for patients to use during consultations with their providers. Breast reconstruction surgeons assessed the quality of the questions in terms of question acceptability, contribution to the informed consent process, and contribution to the shared decision-making process. As a benchmark, the surgeons also assessed a comparison group of questions from credible online sources intended for the general public. Subsequently, the surgeons were asked to indicate which questions they thought were AI-generated.

## MATERIALS AND METHODS

### Generating Questions Using ChatGPT

We prompted ChatGPT to generate lists of questions representative of what breast reconstruction patients might ask. The prompts varied in specificity from “generate a list of questions I can ask my plastic surgeon about breast reconstruction” to “generate a list of questions I can ask my plastic surgeon about the appearance of my breasts after breast reconstruction surgery.” The GPT-3.5 model was queried via its web interface as it is the default model for public use and is available for free.

### Identifying Benchmark Questions From Reputable Sources

Benchmark questions were collected from online websites identified via the Google Search engine with key phrases representative of what breast reconstruction patients might use during preliminary inquiries: “breast reconstruction question list,” “breast reconstruction questions to ask my surgeon,” and “what should I ask my breast reconstruction surgeon.” Only questions from 5 reputable source categories were considered for the study: evidence-based health websites, private breast surgery practices, breast cancer–related nonprofit organizations, government websites, and medical centers.

### Selecting Breast Reconstruction Questions for Assessment

Our queries identified 212 questions from ChatGPT and 407 benchmark questions from reputable websites. Not blinded to the question source, the first author (X.T.G.) eliminated questions that were not related to breast appearance, leaving 101 questions from ChatGPT and 65 benchmark questions. Blinded to the source (ChatGPT versus benchmark set), an experienced plastic surgeon (G.P.R.) further reviewed the questions and eliminated additional nonappearance-related questions, leaving 94 questions from ChatGPT and 64 benchmark questions. The questions were assigned by the first author to 1 of 5 appearance categories (Table 1). Within each category, the first author selected representative questions to minimize duplication of concepts, resulting in final sets of 16 appearance-related questions from ChatGPT and 10 appearance-related benchmark questions.

**Table 1.** Definitions for the 5 Appearance-related Categories of Questions Presented to the Plastic Surgeons

Question Category	Description
Garments	Relates to patient garment wear after breast reconstruction surgery
Nipple reconstruction	Relates to the appearance of a nipple and/or nipple reconstruction after breast reconstruction surgery
Symmetry, size, and shape	Relates to the symmetry, size, and shape of the breasts after breast reconstruction surgery
Healing and scarring	Relates to the healing and scarring of the breasts after breast reconstruction surgery
Expectations	Relates to the patients’ expectations after breast reconstruction surgery

### Assessing Breast Reconstruction Questions

Plastic surgeons from The University of Texas MD Anderson Cancer Center were recruited to assess the questions. Eligibility criteria included having board certification in the United States and expertise in breast reconstruction. The institutional review board (IRB) at The University of Texas at Austin (IRB no. 00004750) determined that this study meets the criteria for exemption from IRB review under 45 CFR 46.104 (2)(ii). Informed consent was obtained electronically from the participants. Participants did not receive compensation.

Questions were presented to participating surgeons via the Qualtrics platform (Qualtrics, Provo, UT). Blinded to the question source (ChatGPT versus benchmark set), participants assessed each question in terms of acceptability, contribution to the informed consent process, and contribution to the shared decision-making process (Table 2).^29^ First, each question was rated as acceptable or unacceptable to ensure that it was understandable and relevant to breast reconstruction. For each acceptable question, the participants reported their agreement on a 4-point Likert scale (1 = strongly disagree, 2 = disagree, 3 = agree, and 4 = strongly agree) that the question would contribute to the informed consent process and again that it would contribute to the shared decision-making process. All items included an optional text box for feedback. The data collection instruments were first tested within the research group to ensure organization and flow.

**Table 2.** Criteria Used to Assess ChatGPT-generated and Benchmark Questions

Criterion	Description
Acceptability	I understand the question and it is relevant to consultation about breast reconstruction
Informed consent	This question would positively contribute to the informed consent process *Informed Consent as defined by the National Cancer Institute:* “A process in which patients are given important information, including possible risks and benefits, about a medical procedure or treatment, genetic testing, or a clinical trial. This is to help them decide if they want to be treated, tested, or take part in the trial. Patients are also given any new information that might affect their decision to continue.”^29^
Shared decision-making	This question would positively contribute to the shared decision-making process *Shared decision-making as defined by the National Cancer Institute:* Shared decision-making is “a process in which both the patient and healthcare professional work together to decide the best plan of care for the patient. When making a shared decision, the patient’s values, goals, and concerns are considered. Shared decision-making helps patients learn more about their health condition, the different testing and treatment options that may be available, and the possible risks and benefits of each option.”^29^

Two months after the initial survey, the surgeons were presented with all 26 questions (with the source hidden) and asked to indicate their agreement on a 4-point Likert scale regarding whether each question was AI-generated.

### Statistical Analyses

Descriptive statistics were computed to summarize surgeon demographics and professional characteristics. Descriptive statistics for survey responses were calculated separately for ChatGPT-generated and benchmark questions. For each set, the analysis included statistics for all questions and for each surgeon individually. A question was considered acceptable if at least 80% of surgeons rated it as such. Positive contributions to the informed consent and shared decision-making processes were defined as median scores of at least 3 on the Likert scale. Similarly, a question was considered to be AI-generated if it received a median score of at least 3. Surgeons’ comments were analyzed to identify potential associations between breast reconstruction topics reflected in the questions and their corresponding ratings.

## RESULTS

### Surgeon Characteristics

Study participants were 5 board-certified surgeons with expertise in breast reconstruction (Table 3). Most participants identified as White, which is the current majority for the profession.^30^ Participants’ mean age was 42 years. The average time participants had been board-certified was 8 years, and they each performed an average of 42 breast reconstructions annually.

**Table 3.** Characteristics of the 5 Board-certified Plastic Surgeons With Expertise in Breast Reconstruction

Characteristic	N
Mean age, y, n (SD)	42.3 (3.5)
Mean no. years with board certification, n (SD)	8.4 (3.2)
Mean no. breast reconstructions performed annually, n (SD)	42.3 (17.8)
Sex assigned at birth, n	
Male	2
Female	2
Declined to answer	1
Gender, n	
Male	2
Female	2
Declined to answer	1
Race, n	
White	4
Declined to answer	1
Ethnicity, n	
Not Hispanic or Latino	4
Declined to answer	1

### Acceptability of Questions Generated by ChatGPT

**Fig. 1.**
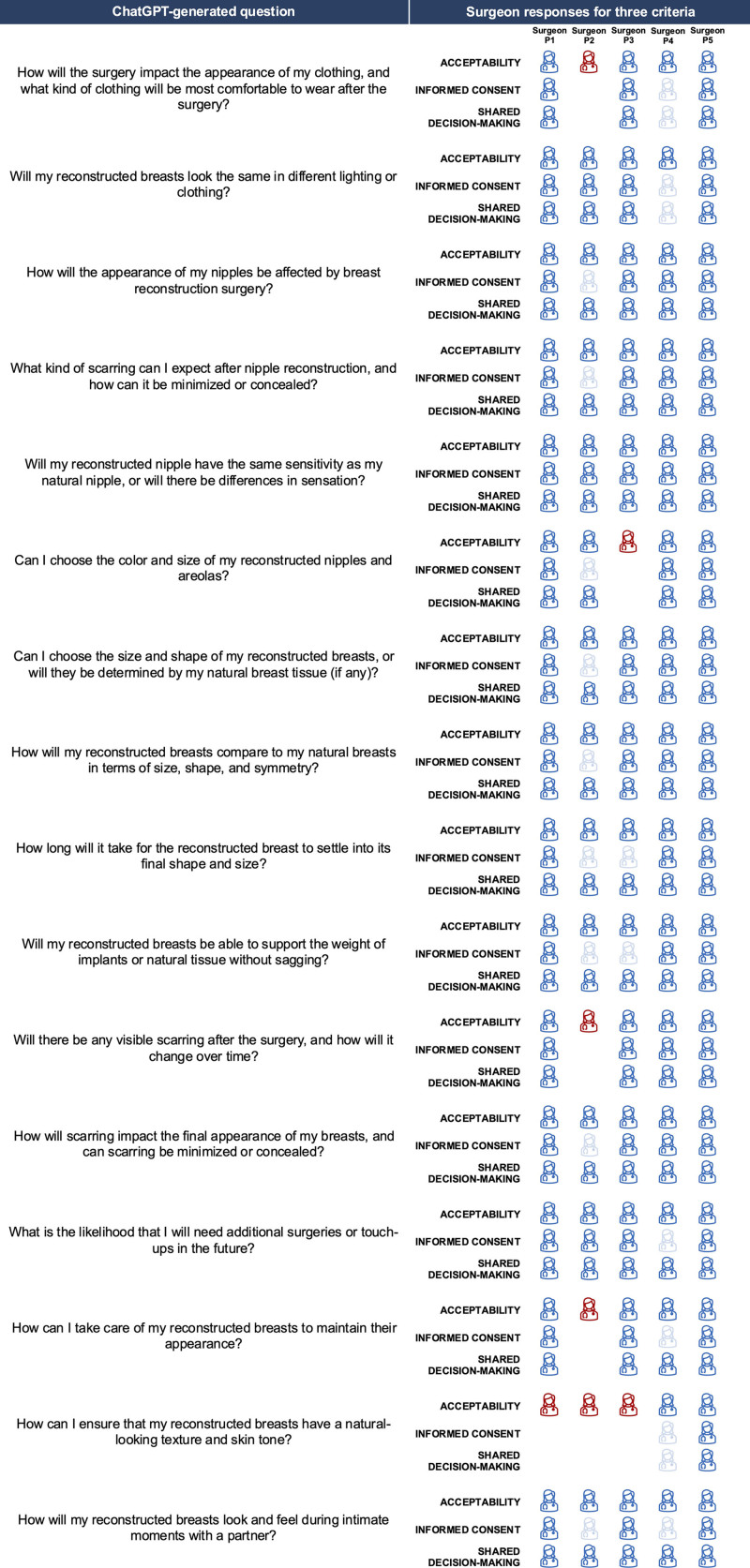
Experienced reconstructive surgeons rated ChatGPT-generated questions concerning the appearance-related outcomes of breast reconstruction in terms of acceptability, likelihood to positively contribute to the informed consent process, and likelihood to positively contribute to the shared decision-making process. Each row of icons corresponds to the ratings of each criterion. Each column of icons corresponds to a single surgeons’ ratings and is denoted by an anonymous identifier (i.e., Surgeon P1). Acceptability was rated as acceptable or not acceptable. Agreement that the question would contribute to the informed consent process and shared decision-making process was rated on a 4-point Likert scale (1 = strongly disagree, 2 = disagree, 3 = agree, 4 = strongly agree). Red icons indicate that a surgeon rated a question as not acceptable. Dark blue icons indicate that a surgeon rated an acceptable question as contributing to informed consent and shared decision-making at the level of 3 or 4. Translucent blue icons indicate that a surgeon rated a question as 1 or 2. Icons for informed consent and shared decision-making have been omitted for questions that the surgeon rated as not acceptable.

Fifteen of the 16 ChatGPT-generated questions assessed were considered acceptable (Fig. 1). The 1 ChatGPT-generated question rated as unacceptable was under the “Expectations” category (*How can I ensure that my reconstructed breasts have a natural-looking texture and skin tone?*). One of the surgeons (participant 1) provided the following feedback: “This is a leading question that imparts all responsibility on the surgeon. I would be hesitant about this one, especially in radiated patients.”

Nine of the 10 benchmark questions were considered acceptable. (**See figure, Supplemental Digital Content 1**, which shows that the assessment of the benchmark questions was comparable to that of the ChatGPT-generated questions, http://links.lww.com/PRSGO/D912.)

### Contribution to the Informed Consent Process of Questions Generated by ChatGPT

Fifteen ChatGPT questions were considered to positively contribute to the informed consent process (Fig. 1). The 1 ChatGPT-generated question rated as not positively contributing to the informed consent process was under the “Expectations” category (*How can I ensure that my reconstructed breasts have a natural-looking texture and skin tone?*).

Differences of opinion among the surgeons emerged. Specifically, 3 questions were rated poorly (disagree or strongly disagree) on their contributions to the informed consent process by more than 1 surgeon: (1) *How long will it take for the reconstructed breast to settle into its final shape and size?*; (2) *Will my reconstructed breasts be able to support the weight of implants or natural tissue without sagging?*; and (3) *How will my reconstructed breasts look and feel during intimate moments with a partner?* Feedback was not provided for these questions.

One surgeon (participant 4) rated 4 questions poorly (strongly disagree) on their contributions to the informed consent process. One question in the “Garments” category—(1) *How will the surgery impact the appearance of my clothing, and what kind of clothing will be most comfortable to wear after the surgery?—*was rated poorly because “clothing or appearance in clothing is not typically discussed in this setting.” Three questions in the “Expectations” category—(1) *How can I take care of my reconstructed breasts to maintain their appearance?*; (2) *How can I ensure that my reconstructed breasts have a natural-looking texture and skin tone?*; and (3) *How will my reconstructed breasts look and feel during intimate moments with a partner?—*were also poorly rated because the topics they refer to are “not typically discussed at this point.”

All 10 benchmark questions were considered to positively contribute to the informed consent process (**Supplemental Digital Content 1,** http://links.lww.com/PRSGO/D912).

### Contribution to the Shared Decision-making Process of Questions Generated by ChatGPT

All 16 ChatGPT questions were considered to positively contribute to the shared decision-making process (Fig. 1).

However, 1 surgeon (participant 4) rated 3 questions poorly (disagree or strongly disagree) on their contributions to the shared decision-making process because they are “not typically discussed” at this stage. These questions were (1) *How will the surgery impact the appearance of my clothing, and what kind of clothing will be most comfortable to wear after the surgery?*; (2) *Will my reconstructed breasts look the same in different lighting or clothing?*; and (3) *How can I ensure that my reconstructed breasts have a natural-looking texture and skin tone?* All benchmark questions were rated as positively contributing to the shared decision-making process (**Supplemental Digital Content 1,** http://links.lww.com/PRSGO/D912).

### Surgeons’ Assessments of Whether Questions Were AI-generated

Most ChatGPT-generated questions (12 of 16) were not identified as AI-generated by the surgeons. (**See figure, Supplemental Digital Content 2**, which displays how ChatGPT-generated questions were not readily identified as AI-generated by the surgeons in this study, http://links.lww.com/PRSGO/D913.)

Moreover, questions that surgeons thought were AI-generated were still often considered to be acceptable (Supplemental Digital Content 3), likely to positively contribute to the informed consent process (Supplemental Digital Content 4), and likely to positively contribute to the shared decision-making process (Supplemental Digital Content 5). (**See figure, Supplemental Digital Content 3**, which displays how surgeons may consider questions to be acceptable for patients to ask in consultation about breast reconstruction even if they think that the question was AI-generated, http://links.lww.com/PRSGO/D914.) (**See figure, Supplemental Digital Content 4**, which displays how surgeons may consider questions to positively contribute to the informed consent process even if they think that the question was AI-generated, http://links.lww.com/PRSGO/D915.) (**See figure, Supplemental Digital Content 5**, which displays how surgeons may consider questions to positively contribute to the shared decision-making process even if they think that the question was AI-generated, http://links.lww.com/PRSGO/D916.)

## DISCUSSION

Experienced reconstructive surgeons rated almost all of the ChatGPT-generated questions concerning appearance-related outcomes of breast reconstruction as acceptable and likely to positively contribute to the informed consent and shared decision-making processes. Most ChatGPT-generated questions were not identified as such by the surgeons, and questions that surgeons thought were AI-generated were still often considered to meet the 3 criteria.

ChatGPT-generated questions pertaining to the “garments,” “symmetry, size, and shape,” and “expectations” categories were scored lower than others with respect to their value for informed consent. Surgeon feedback indicated that garment-related questions received lower ratings because appearance in clothing is not typically discussed in depth during initial consultations. Prior studies on breast cancer survivors’ garment needs and preferences did not explore the merits of discussing expectations for appearance in garments during breast reconstruction consultations.^31,32^ However, these patients consider garment fit and appearance as vital to their well-being and the return to a sense of normalcy after treatment.^31,32^ Future work should focus on the benefits of addressing garment needs in the early stages of breast reconstruction and how this shapes patients’ expectations of what reconstruction may achieve regarding their appearance in clothing.

Surgeons rated some ChatGPT-generated questions in the “symmetry, size, and shape” and “expectations” categories as less likely to positively contribute to the informed consent process due to their focus on aspects of breast reconstruction that are challenging to predict. This disparity may be attributed to the dual purposes of informed consent: promoting patient autonomy and mitigating potential liability concerns. In contrast, surgeons deemed questions addressing unpredictable topics, such as postoperative skin texture and expected outcomes compared with preoperative breasts, as valuable for shared decision-making. Shared decision-making promotes flexibility in patient-provider treatment decisions based on a patient’s values, goals, and concerns. Typically, medical decisions can be mapped onto a 2-dimensional space considering risk levels (ie, low versus high) and certainty (ie, one optimal decision versus multiple less-optimal options).^33^ However, the extent to which patients engage in informed consent and shared decision-making varies depending on their medial situations. Surgeons’ perceived value of these questions may evolve over time and be influenced by factors such as a patient’s healing progress.

Our findings indicate that the use of AI tools, such as ChatGPT, can be encouraged by clinicians to help patients generate questions in preparation for breast reconstruction consultations. Notably, there are several important considerations we would like to highlight. First, breast reconstruction is typically a multistage process that can span years due to factors such as delayed reconstruction or the need for revision procedures.^34^ Patients may have multiple conversations with their surgeon before each procedure. However, the questions examined in this study did not explicitly address the reconstruction timeline. ChatGPT-generated question lists could better target patients’ individual information needs along their reconstruction journey compared with general lists. Nonetheless, the onus is on patients to tailor their prompts for personalized questions and decide when to ask each one. For example, this may include specifying to ChatGPT a timepoint (eg, initial consultation, before a revision).^24^

Additionally, this study did not investigate surgeons’ perceptions of the types of questions posed. However, prior research has found that asking convergent questions (eg, those requesting low-level answers, such as factual information) is as valuable to learning as asking divergent questions.^35,36^ Convergent questions provide insights into an individual’s misconceptions and give experts the opportunity to offer feedback, redirect thinking, and promote knowledge construction.^35,36^ Even encouraging patients to simply ask 3 generic questions about their treatment options, the benefits/harms of each option, and the likelihood of the benefits/harms occurring greatly improves information delivery by providers.^37^ Fostering an environment where patient-initiated discourse is welcomed, regardless of the type of questions asked, may stimulate discussions catering to patients’ needs. If patients can hint at their concerns, providers may be able to have effective interactions, irrespective of how well formulated the questions are.

ChatGPT responses are also influenced by querying approaches, such as iteratively improving initial outputs within a single message thread, as opposed to starting a new thread for each iteration. For example, the 15 acceptable ChatGPT-generated questions had a median Flesch Reading Ease Index^38^ of 59.7 (range, elementary [80.1]–college [31.7]), which falls within the high school range. Users can follow up to prompt ChatGPT to generate questions at their desired readability level.

Currently, research on ChatGPT in healthcare is primarily driven by US-based higher education institutions and funding sources.^39^ Because cross-cultural functionality is difficult to achieve without a diversity of research teams and settings, it is plausible that ChatGPT suggests questions that may not meet the needs and preferences of patients undergoing breast reconstruction outside the United States. Future work should investigate integrating a user’s context and environment, as well as incorporating the perspectives of a more diverse population of surgeons.

Healthcare teams should be ready to discuss with patients the strengths and limitations of AI tools for breast reconstruction consultation preparation. Additionally, breast cancer organizations, which often serve as trusted sources of information, can provide workshops and prompting guides to help patients use AI tools confidently and effectively.

## CONCLUSIONS

The quality of ChatGPT-generated questions related to concerns about appearance is comparable to that of questions sourced from reputable online websites. Patients may benefit from discussion with their providers about the best practices for using AI tools to prepare for consultations. Providers should expect variation in the quality and quantity of content patients assemble when using online tools. More research is needed on how AI tools can support and personalize patients’ consultation preparation.

## DISCLOSURES

The authors have no financial interest to declare in relation to the content of this article. Gonzalez received funding from the National Institutes of Health fellowship (T32 EB007507).

## ACKNOWLEDGMENTS

The authors thank participants for volunteering their time to complete our study. We appreciate the guidance of the Multidisciplinary Breast Reconstruction Research Program’s Advisory Council, chaired by Dr. Michelle C. Fingeret. The authors thank Dawn Chalaire, Associate Director, Research Medical Library, for editing this article.

## Supplementary Material


