# Supplementary material for: Assessment of Appearance-related Questions About Breast Reconstruction Generated by Chat Generative Pre-trained Transformer
Source: Plast Reconstr Surg Glob Open. 2025 Mar 21;13(3):e6625. doi: 10.1097/GOX.0000000000006625 (PMC11927646; doi:10.1097/GOX.0000000000006625)
Supplement: Supplementary file 1 [file gox-13-e6625-s001.pdf]

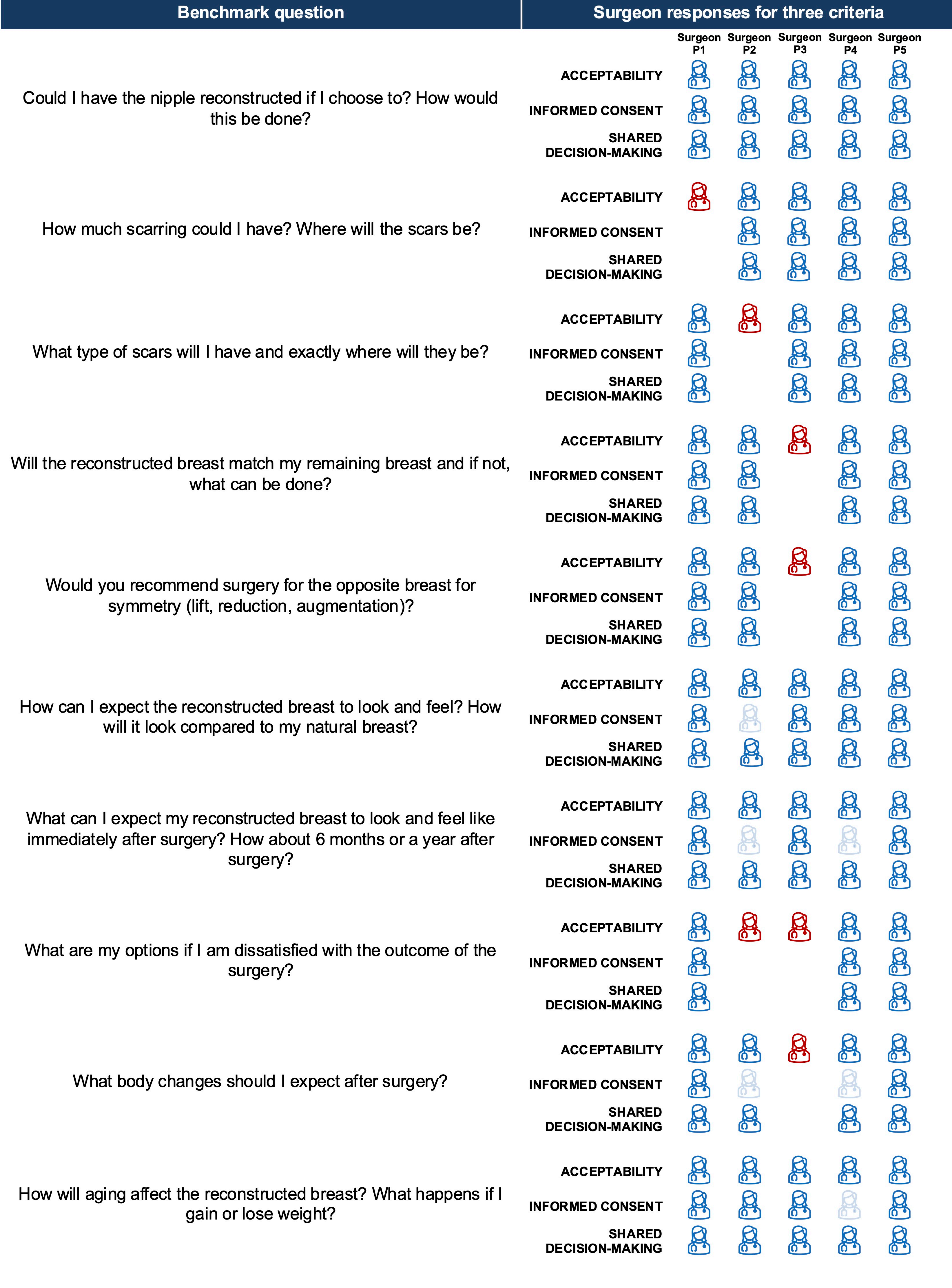

The assessment of the benchmark questions was comparable to that of the ChatGPT-generated questions. Surgeons' ratings for the ten benchmark questions are shown according to three criteria: acceptability, agreement that the question would contribute to the informed-consent process, and agreement that the question would contribute to the shared decision-making process. Each row of icons corresponds to the ratings for each criterion. Each column of icons corresponds to a single surgeon's ratings and is denoted by an anonymous identifier (i.e., Surgeon P1, etc.).

Acceptability was rated as acceptable or not acceptable. Agreement that the question would contribute to the informed consent process and shared decision-making process was rated on a four-point Likert scale (1 = strongly disagree, 2 = disagree, 3 = agree, 4 = strongly agree).

Red icons indicate a surgeon rated a question as not acceptable. Dark-blue icons indicate a surgeon rated an acceptable question as contributing to informed consent and shared decision making at the level of 3 or 4. Translucent-blue icons indicate a surgeon rated a question as 1 or 2. Icons for informed consent and shared decision making have been omitted for questions that the surgeon rated as not acceptable.
