# Supplementary material for: Assessment of Appearance-related Questions About Breast Reconstruction Generated by Chat Generative Pre-trained Transformer
Source: Plast Reconstr Surg Glob Open. 2025 Mar 21;13(3):e6625. doi: 10.1097/GOX.0000000000006625 (PMC11927646; doi:10.1097/GOX.0000000000006625)
Supplement: Supplementary file 2 [file gox-13-e6625-s002.pdf]

All Surgeons

	Surgeons think AI-generated	Surgeons think not AI-generated
ChatGPT	4	12
Benchmark	2	8

Surgeon P1

	Surgeon thinks AI-generated	Surgeon thinks not AI-generated
ChatGPT	3	13
Benchmark	4	6

Surgeon P2

	Surgeon thinks AI-generated	Surgeon thinks not AI-generated
ChatGPT	6	10
Benchmark	3	7

Surgeon P3

	Surgeon thinks AI-generated	Surgeon thinks not AI-generated
ChatGPT	4	12
Benchmark	3	7

Surgeon P4

	Surgeon thinks AI-generated	Surgeon thinks not AI-generated
ChatGPT	6	10
Benchmark	3	7

Surgeon P5

	Surgeon thinks AI-generated	Surgeon thinks not AI-generated
ChatGPT	0	16
Benchmark	0	10

ChatGPT-generated questions were not readily identified as AI-generated by the surgeons in this study. Pooled and individual-surgeon responses as to whether they believed that the 16 ChatGPT-generated questions and 10 benchmark questions were AI-generated are shown. Responses were made using a four-point Likert scale (1 = strongly disagree, 2 = disagree, 3 = agree, 4 = strongly agree). For the pooled surgeon responses, if the median rating was at least 3, we interpreted that to mean that the surgeons thought that the question was AI-generated.
