# Supplementary material for: Assessment of Appearance-related Questions About Breast Reconstruction Generated by Chat Generative Pre-trained Transformer
Source: Plast Reconstr Surg Glob Open. 2025 Mar 21;13(3):e6625. doi: 10.1097/GOX.0000000000006625 (PMC11927646; doi:10.1097/GOX.0000000000006625)
Supplement: Supplementary file 3 [file gox-13-e6625-s003.pdf]

All Surgeons

	Acceptable	Not acceptable
Surgeons think AI-generated	3	1
Surgeons think not AI-generated	12	0

Surgeon P1

	Acceptable	Not acceptable
Surgeon thinks AI-generated	3	0
Surgeon thinks not AI-generated	12	1

Surgeon P2

	Acceptable	Not acceptable
Surgeon thinks AI-generated	4	2
Surgeon thinks not AI-generated	8	2

Surgeon P3

	Acceptable	Not acceptable
Surgeon thinks AI-generated	3	1
Surgeon thinks not AI-generated	11	1

Surgeon P4

	Acceptable	Not acceptable
Surgeon thinks AI-generated	6	0
Surgeon thinks not AI-generated	10	0

Surgeon P5

	Acceptable	Not acceptable
Surgeon thinks AI-generated	0	0
Surgeon thinks not AI-generated	16	0

Surgeons may consider questions to be acceptable for patients to ask in consultation about breast reconstruction even if they think that the question was AI-generated. Pooled and individual surgeon ratings for 16 ChatGPT-generated questions as to whether they thought the questions were AI-generated and met the acceptability criterion are reported.

Responses as to whether questions were believed to be AI-generated were made using a four-point Likert scale (1 = strongly disagree, 2 = disagree, 3 = agree, 4 = strongly agree). For the pooled surgeon responses, if the median rating was at least 3, we interpreted that to mean that the surgeons thought that the question was AI-generated. Acceptability was rated as acceptable or not acceptable. For pooled responses, a question was considered acceptable if at least 4 of the 5 surgeons rated it as acceptable.
