# Supplementary material for: Assessment of Appearance-related Questions About Breast Reconstruction Generated by Chat Generative Pre-trained Transformer
Source: Plast Reconstr Surg Glob Open. 2025 Mar 21;13(3):e6625. doi: 10.1097/GOX.0000000000006625 (PMC11927646; doi:10.1097/GOX.0000000000006625)
Supplement: Supplementary file 5 [file gox-13-e6625-s005.pdf]

All Surgeons

	Positively contributes to shared decision making	Does not positively contribute to shared decision making
**Surgeon thinks AI-generated**	4	0
**Surgeon thinks not AI-generated**	12	0

Surgeon P1

	Positively contributes to shared decision making	Does not positively contribute to shared decision making
**Surgeon thinks AI-generated**	3	0
**Surgeon thinks not AI-generated**	12	0

Surgeon P2

	Positively contributes to shared decision making	Does not positively contribute to shared decision making
**Surgeon thinks AI-generated**	4	0
**Surgeon thinks not AI-generated**	8	0

Surgeon P3

	Positively contributes to shared decision making	Does not positively contribute to shared decision making
**Surgeon thinks AI-generated**	3	0
**Surgeon thinks not AI-generated**	11	0

Surgeon P4

	Positively contributes to shared decision making	Does not positively contribute to shared decision making
**Surgeon thinks AI-generated**	4	2
**Surgeon thinks not AI-generated**	9	1

Surgeon P5

	Positively contributes to shared decision making	Does not positively contribute to shared decision making
**Surgeon thinks AI-generated**	0	0
**Surgeon thinks not AI-generated**	16	0

Surgeons may consider questions to positively contribute to the shared decision-making process even if they think that the question was AI-generated. Pooled and individual surgeon ratings for 16 ChatGPT-generated questions as to whether they thought the questions were AI-generated and positively contributed to the shared decision-making process are reported.

Responses as to whether questions were believed to be AI-generated and contribute to the shared decision-making process were made using a four-point Likert scale (1 = strongly disagree, 2 = disagree, 3 = agree, 4 = strongly agree). For the pooled surgeon responses, if the median rating was at least a 3, we interpreted that to mean that the surgeons thought that the question was AI-generated. The same median cutoff was considered to determine positive contribution to the shared decision-making process.
